# Supplementary material for: Modeling the impact of vaccination strategies for nursing homes in the context of increased SARS-CoV-2 community transmission and variants
Source: medRxiv. 2021 Oct 26:2021.10.25.21265493. Preprint. [Version 1] doi: 10.1101/2021.10.25.21265493 (PMC8562554; doi:10.1101/2021.10.25.21265493)
Supplement: 1 [file NIHPP2021.10.25.21265493V1-supplement-1.pdf]

medRxiv preprint doi: https://doi.org/10.1101/2021.10.25.21265493; this version posted October 26, 2021. The copyright holder for this preprint (which was not certified by peer review) is the author/funder, who has granted medRxiv a license to display the preprint in perpetuity.
This article is a US Government work. It is not subject to copyright under 17 USC 105 and is also made available for use under a CC0 license.

Supplement

Table S1

**Parameter**	**Values****
R_0	6 (Delta estimate) [1,2]
Probability of infection per infectious contact	0.0372
Daily contacts staff-staff	2 [3]
Daily contacts residents - staff	6 [3,4]
Daily contacts staff - residents	6 - 12 [3]
Daily contacts residents - residents (non-roommates)	6
Daily visitor probability	0.1
Latent period (days)	3-5 [5]
Proportion of unvaccinated staff asymptomatic	0.4 [6,7]
Proportion of unvaccinated residents asymptomatic	0.2 [8-10]
Duration of pre-symptomatic transmission (days)	2 [5,7,11]
Time in infectious compartment (days); infectiousness dependent on viral load	14 [12]

medRxiv preprint doi: https://doi.org/10.1101/2021.10.25.21265493; this version posted October 26, 2021. The copyright holder for this preprint (which was not certified by peer review) is the author/funder, who has granted medRxiv a license to display the preprint in perpetuity. This article is a US Government work. It is not subject to copyright under 17 USC 105 and is also made available for use under a CC0 license.

Reduction in force of infection per contact from PPE	95% [13]
COVID-19 mortality (daily)	0.02
Mean peak viral load (copies/mL)	10^8 [14]
Limit of detection - rapid antigen test (copies/mL)	10^5 [15-18]
Antigen test specificity	1
Viral load threshold for infectiousness (copies/mL)	10^4
Viral load threshold for high infectiousness (copies/mL)	10^7
Time until effect of vaccine dose after vaccination (days)	14 days
Staff daily probability of community infection	0.001 - 0.003 [19]
Resident vaccination coverage	80% [20]
Staff vaccination coverage	40% - 100%
2 dose vaccine efficacy against infection (staff)	70% [21, 22]
2 dose vaccine efficacy against infection (residents)	50% - 70% [21, 22]
2 dose vaccine efficacy against progression to symptoms (staff)*	70% [22]

medRxiv preprint doi: https://doi.org/10.1101/2021.10.25.21265493; this version posted October 26, 2021. The copyright holder for this preprint (which was not certified by peer review) is the author/funder, who has granted medRxiv a license to display the preprint in perpetuity. This article is a US Government work. It is not subject to copyright under 17 USC 105 and is also made available for use under a CC0 license.

2 dose vaccine efficacy against progression to symptoms (resident)*	60% - 66.67% [22]
2 dose vaccine efficacy against symptomatic disease* (staff)	90% [22]
2 dose vaccine efficacy against symptomatic disease* (residents)	80% - 90% [21, 22]
vaccine efficacy against infectiousness (residents and staff)	50% - 90%
Booster dose vaccine efficacy against infection (residents, takes 2 weeks to take effect)	60% - 90%

* VE against symptomatic disease = 1 - (1 - VE against infection)*(1-VE against progression to symptoms)

** If no references are listed, values are assumed

medRxiv preprint doi: https://doi.org/10.1101/2021.10.25.21265493; this version posted October 26, 2021. The copyright holder for this preprint
(which was not certified by peer review) is the author/funder, who has granted medRxiv a license to display the preprint in perpetuity.
This article is a US Government work. It is not subject to copyright under 17 USC 105 and is also made available
for use under a CC0 license.

Figure S1

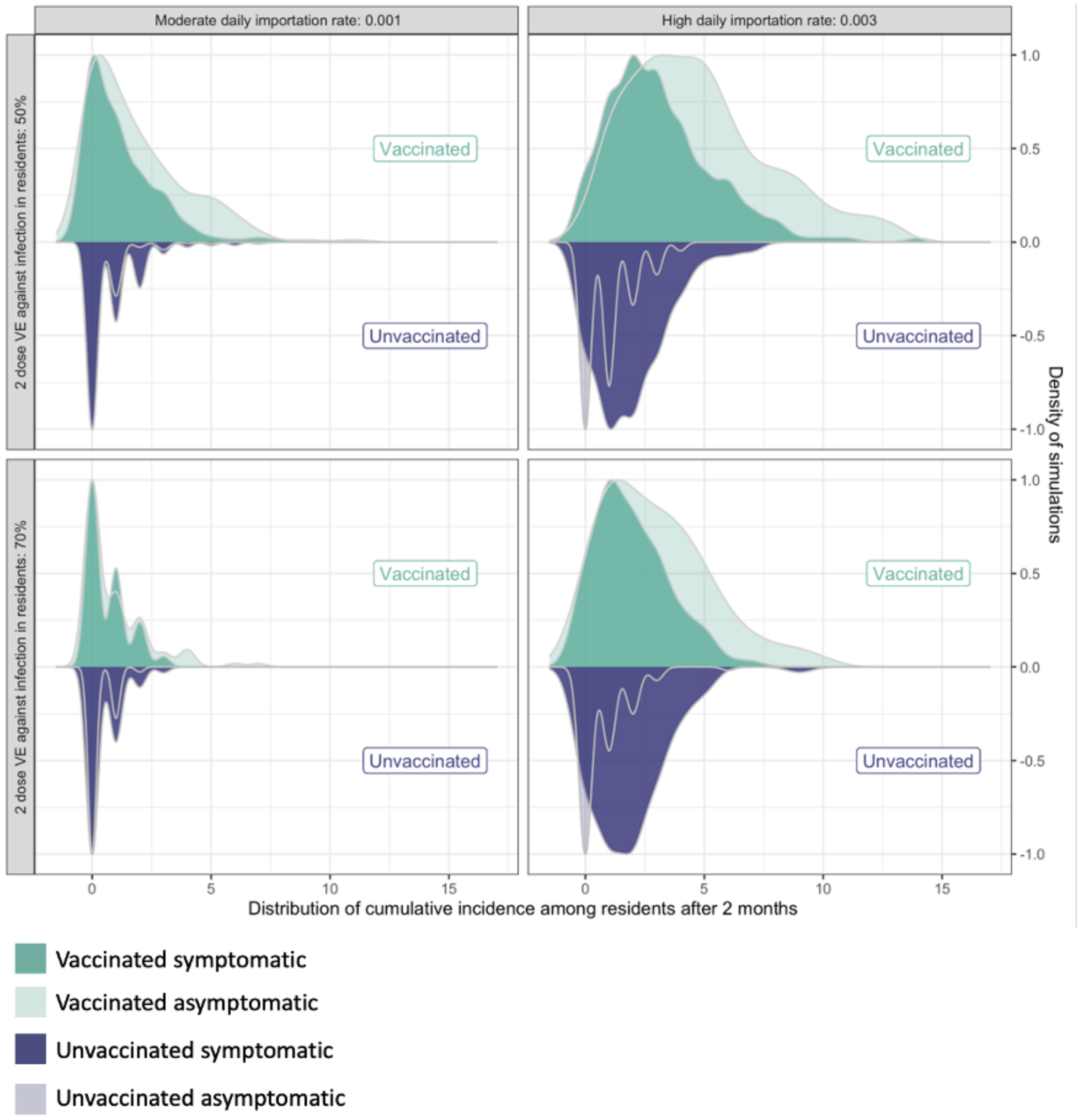

Distribution of cumulative cases across 100 simulations over a two-month period disaggregated by symptom and vaccination status. Over this period there are an average of 217 unique residents in the nursing home; 80% of residents are fully vaccinated (including those who are admitted during the simulation) and 60% of staff are fully vaccinated.

medRxiv preprint doi: https://doi.org/10.1101/2021.10.25.21265493; this version posted October 26, 2021. The copyright holder for this preprint (which was not certified by peer review) is the author/funder, who has granted medRxiv a license to display the preprint in perpetuity. This article is a US Government work. It is not subject to copyright under 17 USC 105 and is also made available for use under a CC0 license.

Figure S2

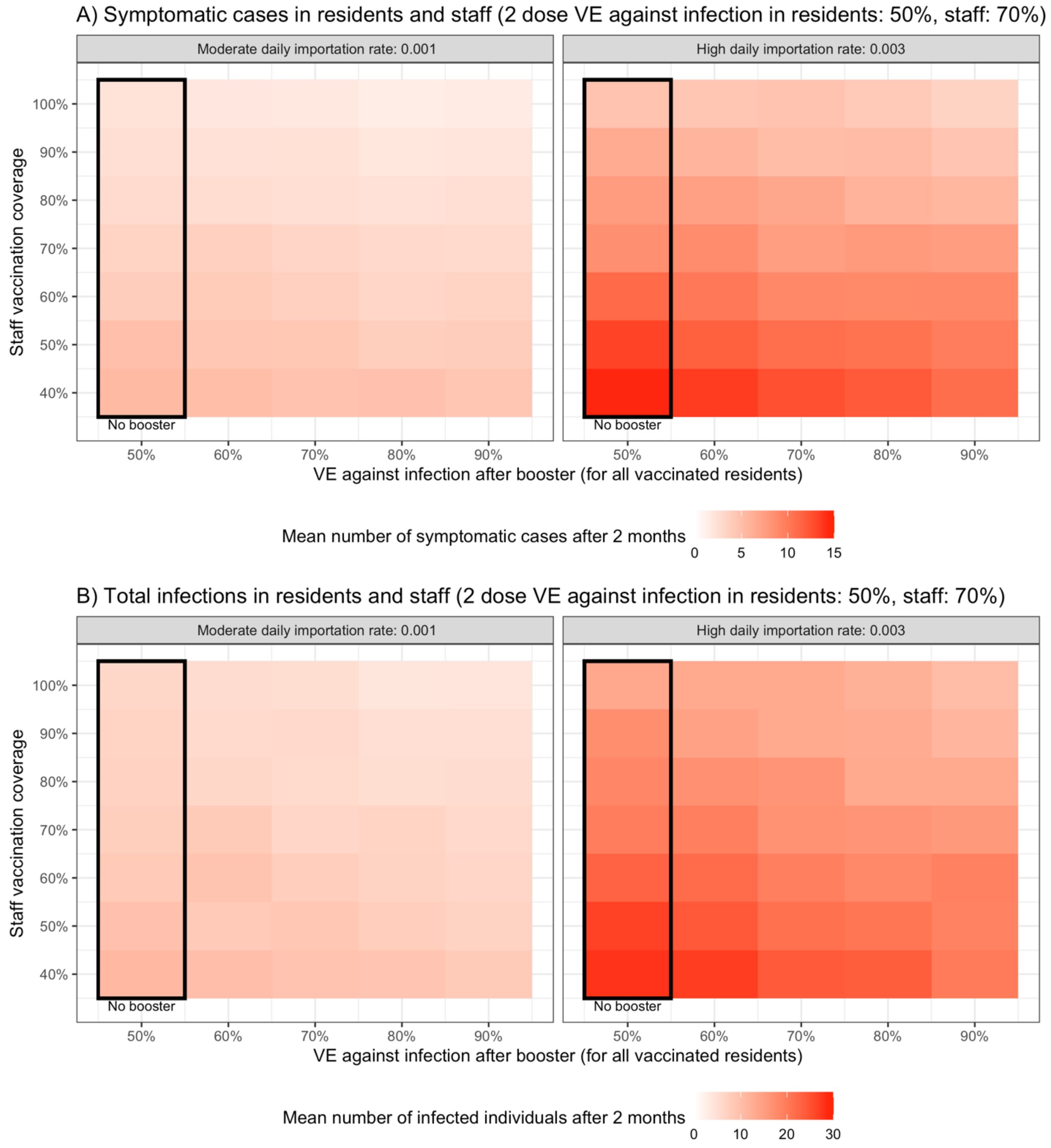

Average cumulative number across 100 simulations of A) symptomatic residents and staff and B) infected residents and staff (symptomatic and asymptomatic) after 2 months, varying staff coverage (rows), booster VE (columns), and staff importation rates (panels)

medRxiv preprint doi: https://doi.org/10.1101/2021.10.25.21265493; this version posted October 26, 2021. The copyright holder for this preprint
(which was not certified by peer review) is the author/funder, who has granted medRxiv a license to display the preprint in perpetuity.
This article is a US Government work. It is not subject to copyright under 17 USC 105 and is also made available
for use under a CC0 license.

Figure S3

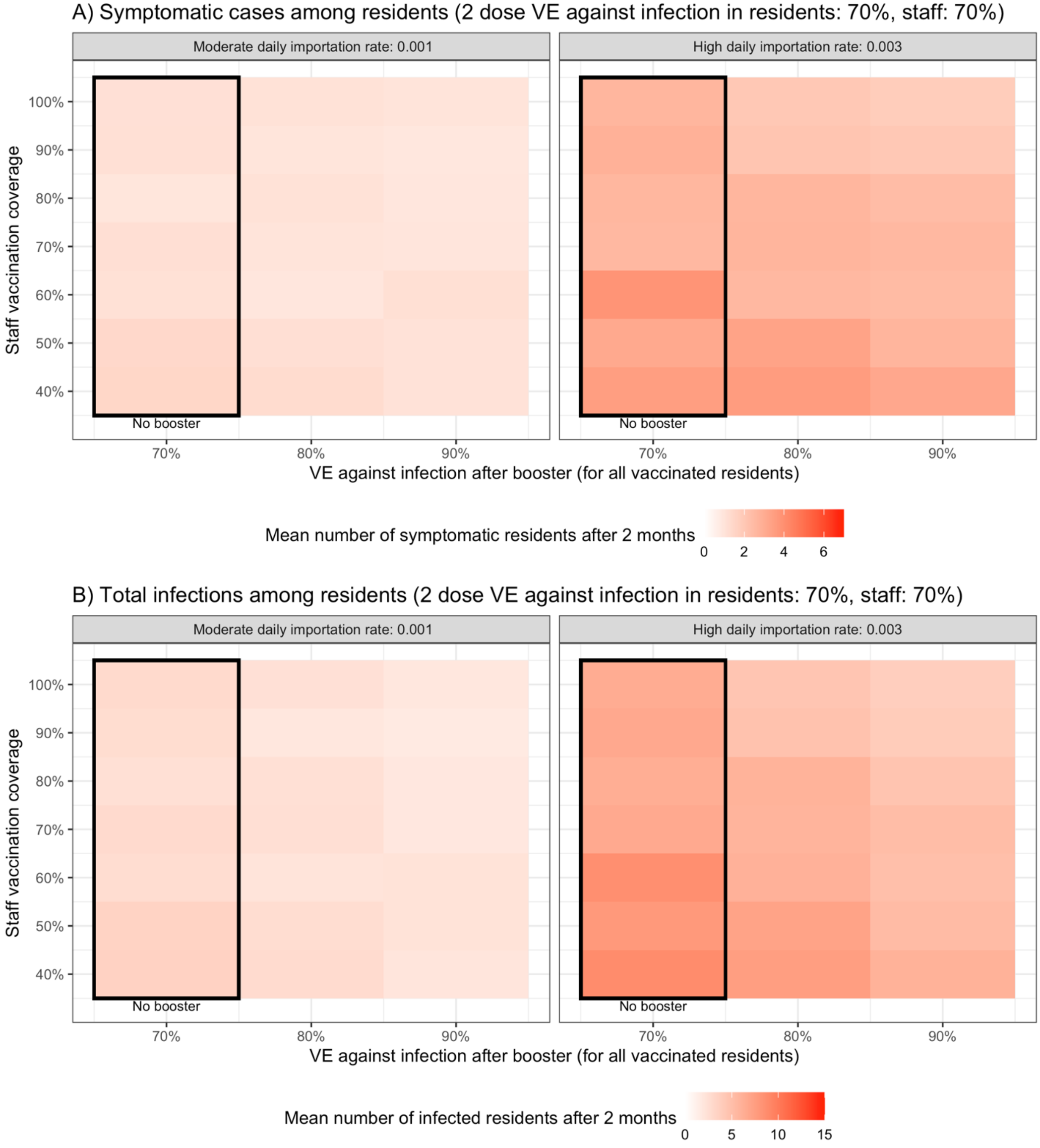

Average cumulative number across 100 simulations of A) symptomatic residents and B) infected residents (symptomatic and asymptomatic) after 2 months, varying staff coverage (rows), booster VE (columns), and staff importation rates (panels) in simulations with higher VE against infection among residents than in the baseline simulations

medRxiv preprint doi: https://doi.org/10.1101/2021.10.25.21265493; this version posted October 26, 2021. The copyright holder for this preprint (which was not certified by peer review) is the author/funder, who has granted medRxiv a license to display the preprint in perpetuity. This article is a US Government work. It is not subject to copyright under 17 USC 105 and is also made available for use under a CC0 license.

Figure S4

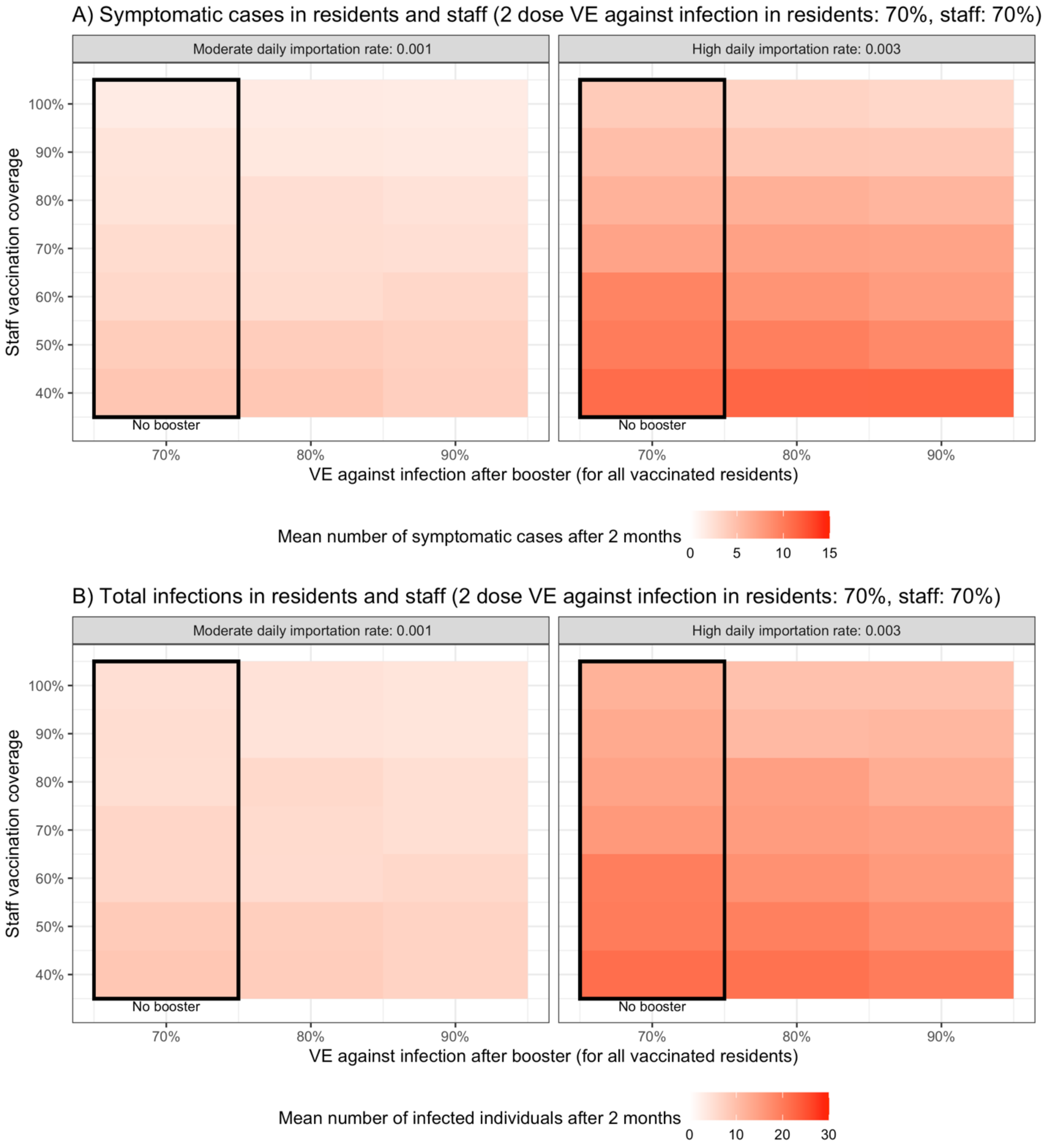

Average cumulative number across 100 simulations of A) symptomatic residents and staff and B) infected residents and staff (symptomatic and asymptomatic) after 2 months, varying staff coverage (rows), booster VE (columns), and staff importation rates (panels) in simulations with higher VE against infection among residents than in the baseline simulations

medRxiv preprint doi: https://doi.org/10.1101/2021.10.25.21265493; this version posted October 26, 2021. The copyright holder for this preprint (which was not certified by peer review) is the author/funder, who has granted medRxiv a license to display the preprint in perpetuity.
This article is a US Government work. It is not subject to copyright under 17 USC 105 and is also made available for use under a CC0 license.

Figure S5

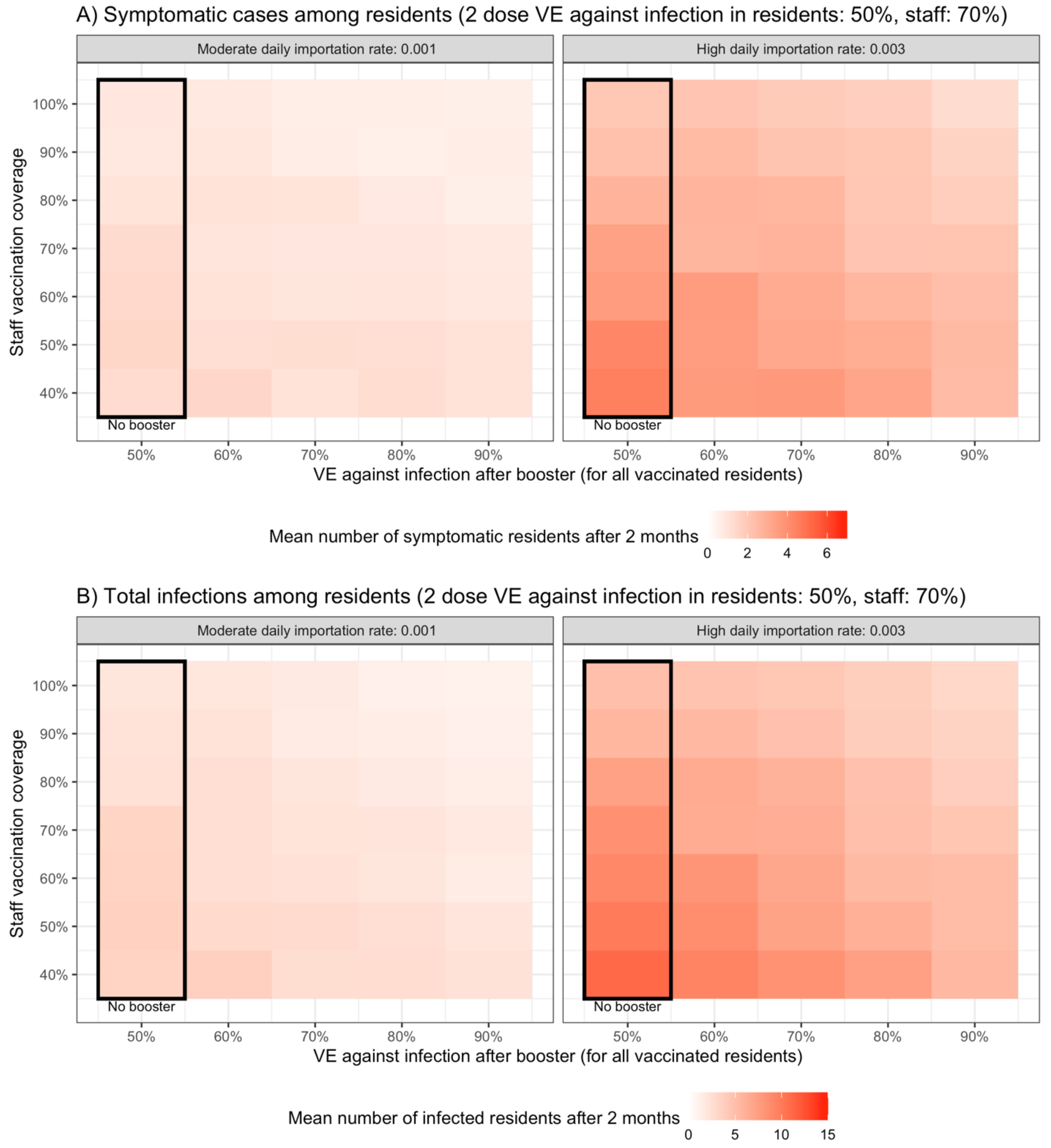

Average cumulative number across 100 simulations of A) symptomatic residents and B) infected residents (symptomatic and asymptomatic) after 2 months, varying staff coverage (rows), booster VE (columns), and staff importation rates (panels) in simulations with higher VE against infectiousness among residents than in the baseline simulations (90% vs. 50%)

medRxiv preprint doi: https://doi.org/10.1101/2021.10.25.21265493; this version posted October 26, 2021. The copyright holder for this preprint (which was not certified by peer review) is the author/funder, who has granted medRxiv a license to display the preprint in perpetuity.
This article is a US Government work. It is not subject to copyright under 17 USC 105 and is also made available for use under a CC0 license.

Figure S6

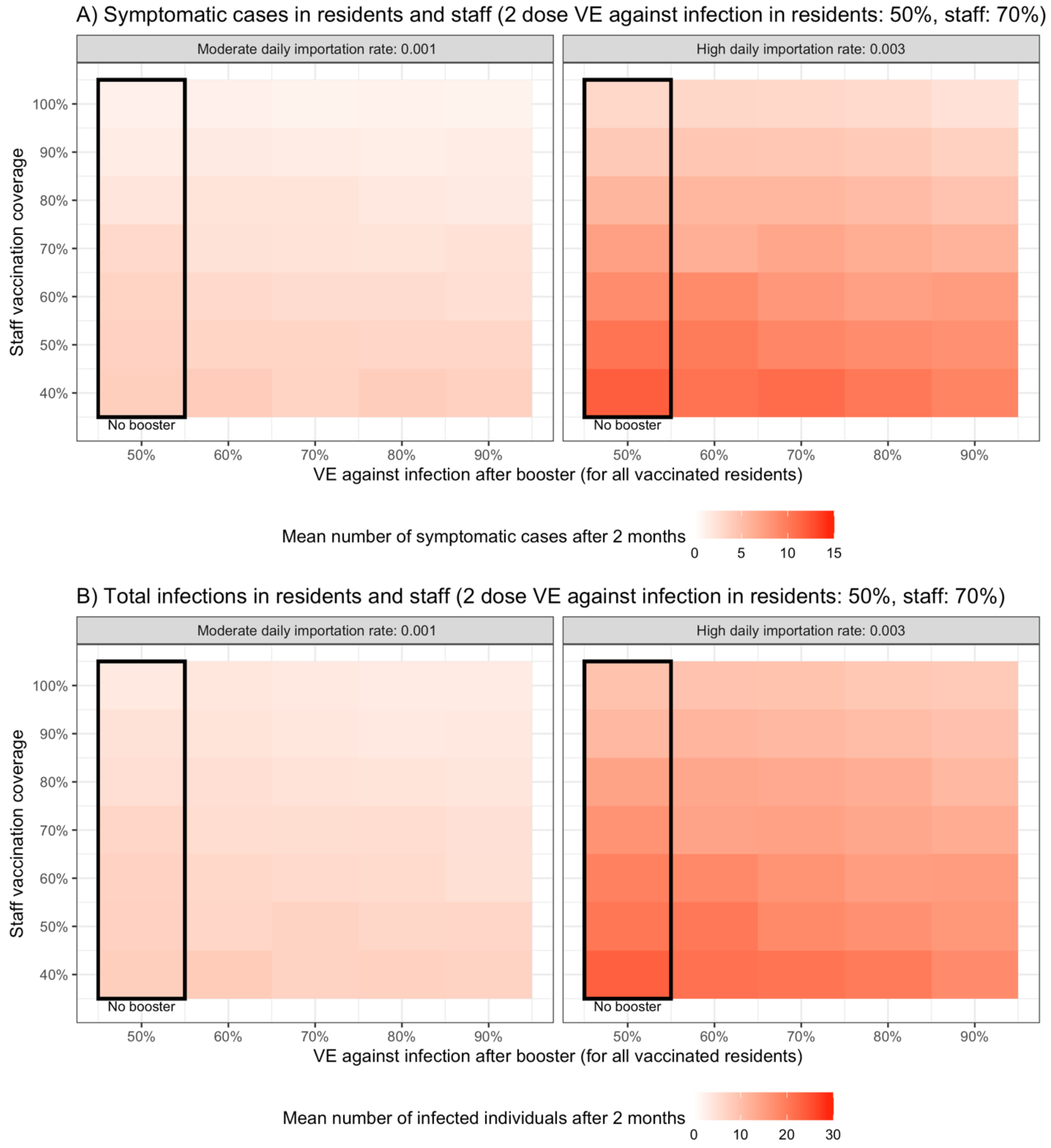

Average cumulative number across 100 simulations of A) symptomatic residents and staff and B) infected residents and staff (symptomatic and asymptomatic) after 2 months, varying staff coverage (rows), booster VE (columns), and staff importation rates (panels) in simulations with higher VE against infectiousness among residents than in the baseline simulations (90% vs. 50%)

medRxiv preprint doi: https://doi.org/10.1101/2021.10.25.21265493; this version posted October 26, 2021. The copyright holder for this preprint (which was not certified by peer review) is the author/funder, who has granted medRxiv a license to display the preprint in perpetuity.
This article is a US Government work. It is not subject to copyright under 17 USC 105 and is also made available for use under a CC0 license.

References

1. CDC. COVID Data Tracker. 2020. Available at: https://covid.cdc.gov/covid-data-tracker/. Accessed 5 September 2021.

2. Burki TK. Lifting of COVID-19 restrictions in the UK and the Delta variant. Lancet Respir Med **2021**; 9:e85.

3. Anglo R. Chief Clinical Officer, Chelsea Jewish Life Care, Personal communication, Jun 10, 2020.

4. Samore M. Professor of Internal Medicine, University of Utah. Personal communication, Dec 08, 2020.

5. Bar-On YM, Flamholz A, Phillips R, Milo R. SARS-CoV-2 (COVID-19) by the numbers. Elife 2020; 9. Available at: https://www.ncbi.nlm.nih.gov/pmc/articles/PMC7224694/. Accessed 27 September 2020.

6. Oran DP, Topol EJ. Prevalence of Asymptomatic SARS-CoV-2 Infection : A Narrative Review. Ann Intern Med 2020; 173:362–367.

7. Johansson MA, Quandelacy TM, Kada S, et al. SARS-CoV-2 Transmission From People Without COVID-19 Symptoms. JAMA Netw Open 2021; 4:e2035057–e2035057.

8. CDC. Healthcare Workers. 2020. Available at: https://www.cdc.gov/coronavirus/2019-ncov/hcp/planning-scenarios.html. Accessed 17 January 2021.

9. Lennon NJ, Bhattacharyya RP, Mina MJ, et al. Comparison of viral levels in individuals with or without symptoms at time of COVID-19 testing among 32,480 residents and staff of nursing homes and assisted living facilities in Massachusetts. Public and Global Health. 2020; Available at: https://www.medrxiv.org/content/10.1101/2020.07.20.20157792v1.abstract.

10. Byambasuren O, Cardona M, Bell K, Clark J, McLaws M-L, Glasziou P. Estimating the extent of asymptomatic COVID-19 and its potential for community transmission: systematic review and meta-analysis. Available at: http://dx.doi.org/10.1101/2020.05.10.20097543.

11. Li Q, Guan X, Wu P, et al. Early Transmission Dynamics in Wuhan, China, of Novel Coronavirus-Infected Pneumonia. N Engl J Med 2020; 382:1199–1207.

12. Holmdahl I, Kahn R, Hay JA, Buckee CO, Mina MJ. Estimation of Transmission of COVID-19 in Simulated Nursing Homes With Frequent Testing and Immunity-Based Staffing. JAMA Open 2021; 4:e2110071–e2110071.

13. Livingston E, Desai A, Berkwits M. Sourcing Personal Protective Equipment During the COVID-19 Pandemic. JAMA 2020; 323:1912–1914.

14. Wölfel R, Corman VM, Guggemos W, et al. Virological assessment of hospitalized patients with COVID-2019. Nature 2020; 581:465–469.

medRxiv preprint doi: https://doi.org/10.1101/2021.10.25.21265493; this version posted October 26, 2021. The copyright holder for this preprint (which was not certified by peer review) is the author/funder, who has granted medRxiv a license to display the preprint in perpetuity. This article is a US Government work. It is not subject to copyright under 17 USC 105 and is also made available for use under a CC0 license.

15. Larremore DB, Wilder B, Lester E, et al. Test sensitivity is secondary to frequency and turnaround time for COVID-19 surveillance. medRxiv 2020; :2020.06.22.20136309.

16. Butler DJ, Mozsary C, Meydan C, et al. Shotgun Transcriptome and Isothermal Profiling of SARS-CoV-2 Infection Reveals Unique Host Responses, Viral Diversification, and Drug Interactions. bioRxiv 2020; Available at: http://dx.doi.org/10.1101/2020.04.20.048066.

17. Dao Thi VL, Herbst K, Boerner K, et al. A colorimetric RT-LAMP assay and LAMP-sequencing for detecting SARS-CoV-2 RNA in clinical samples. Sci Transl Med 2020; 12. Available at: http://dx.doi.org/10.1126/scitranslmed.abc7075.

18. Meyerson NR, Yang Q, Clark SK, et al. A community-deployable SARS-CoV-2 screening test using raw saliva with 45 minutes sample-to-results turnaround. Infectious Diseases (except HIV/AIDS). 2020; Available at: https://www.medrxiv.org/content/10.1101/2020.07.16.20150250v1.abstract.

19. CDC. National Healthcare Safety Network COVID-19 Data Dashboard. 2021. Available at: https://www.cdc.gov/nhsn/covid19/ltc-report-overview.html. Accessed 5 September 2021.

20. Centers for Medicare & Medicaid Services Data. Available at: https://www.google.com/url?q=https://data.cms.gov/covid-19/covid-19-nursing-home-data&sa=D&source=editors&ust=1630867449026000&usg=AOvVaw3FTiBsOErdqOXvIuNCr4Cz. Accessed 5 September 2021.

21. Nanduri S. Effectiveness of Pfizer-BioNTech and Moderna Vaccines in Preventing SARS-CoV-2 Infection Among Nursing Home Residents Before and During Widespread Circulation of the SARS-CoV-2 B.1.617.2 (Delta) Variant — National Healthcare Safety Network, March 1–August 1, 2021. MMWR Morb Mortal Wkly Rep 2021; 70. Available at: https://www.cdc.gov/mmwr/volumes/70/wr/mm7034e3.htm. Accessed 5 September 2021.

22. Higdon MM, Wahl B, Jones CB, et al. A systematic review of COVID-19 vaccine efficacy and effectiveness against SARS-CoV-2 infection and disease. medRxiv 2021; :2021.09.17.21263549.
